# Research on Temperature Field Distribution in a Frame Mold during Autoclave Process

**DOI:** 10.3390/ma13184020

**Published:** 2020-09-10

**Authors:** Ning Han, Luling An, Longxin Fan, Leilei Hua, Guoqiang Gao

**Affiliations:** College of Mechanical and Electrical Engineering, Nanjing University of Aeronautics and Astronautics, Nanjing 210016, China; hanning@nuaa.edu.cn (N.H.); fanlx@nuaa.edu.cn (L.F.); hualei_nuaa@163.com (L.H.); nuaaall@126.com (G.G.)

**Keywords:** autoclave, mold, thermal analysis, CFD simulation

## Abstract

The success of an autoclave process is related to the temperature characteristics of the mold. An inhomogeneous temperature field in the mold affects the quality of composite parts, which may lead to residual stress, voids, and other manufacturing defects of composite parts. In order to meet high-quality production demands, the temperature field in a mold should be investigated precisely. The temperature distribution in a large frame mold is critically evaluated in this work. Then, a method to control the temperature distribution in a large frame mold is proposed. A computational fluid dynamics (CFD) model of the autoclave process is developed to predict the temperature evolution of the large frame mold. The model is validated by experimental results, which shows good agreement with a relative difference of 5.92%. The validated CFD model is then applied to analyze the temperature distribution characters in the mold with different control conditions. The results show that the temperature difference decreases by 13.3% when the mold placement angle is changed from 180 to 168°.

## 1. Introduction

The autoclave molding technology is widely used in the aircraft and space industry to produce high-performance composite parts [1,2]. Mechanical properties of composite materials mainly depend on their curing temperature and pressure in the autoclave [3,4,5]. Thermosetting composites generally undergo three stages in the curing process with the increase in curing temperature, namely, liquid stage, rubber stage, and solid stage [6,7]. Generally, the composite changes from the liquid stage to rubber stage at the first increasing temperature stage in the autoclave process. The temperature gradient of composite will cause uneven resin flow and void formation at this stage [8,9]. Therefore, it is vital to reduce the temperature gradient of composite part for improving the molding quality. There are two main heat sources for the composite in the curing process. One is the convection heat transfer of heated air, and the other is the heat conduction between the mold and the composite [10,11,12]. Therefore, reducing the temperature gradient in the mold has a positive effect on the molding quality of composites.

The temperature field of a composite part is a critical factor influencing the curing quality of a composite product [13,14,15,16]. Researchers have extensively investigated the temperature field of composite parts during the autoclave process by means of simulation and experiments. Bogetti and Giliespie [17] built a process model for the different boundary conditions and geometry of the composite part using the two-dimensional finite element method. The model presented the temperature gradient in the thick composite part for different curing cycles. Struzziero and Skordos [18] presented a multi-objective optimization method, which aims to both minimize the curing process duration and maximum temperature overshoot within the part. White and Kim [19] developed a two-stage curing method to solve problems of thermal spiking and nonuniform consolidation in thick laminates. They demonstrated that the new curing method reduced the void content.

Compared with the numerous studies on the effect of curing circle temperature on the temperature field of composite parts, little attention has been paid to the influence of mold on the temperature field of the part. Wang et al. [20] combined the numerical model with the genetic algorithm to optimize the design parameters of the substructure of the mold. Xie et al. [21] performed a simulation for analyzing the heat transfer in the mold during an autoclave curing cycle. An improved curing process was proposed to achieve better uniformity of the temperature field of the composite product. Weber et al. [22] summarized the essential factors influencing the convective heat transfer coefficient in the mold panel. They proposed an approach using shift factors in combination with a measured reference curve, which was introduced into a thermal simulation. However, the shift factors presented were only valid for simple molds of different sizes. Chen et al. [23] conducted a simulation of mold in the autoclave, which focused on the study of boundary layer grids to make the simulation results more accurate. Kluge et al. [24] investigated the temperature distribution in the industrial mold in an autoclave. The results indicated that positions on the upstream side of the tool are heated faster than the downstream positions. The temperature difference in the mold cannot be eliminated due to the characteristics of heat transfer in an autoclave process [25]. Therefore, it is essential to minimize the temperature difference in the mold.

The objective of this paper was to critically assess the temperature field in a large frame mold and improve the temperature distribution uniformity in the mold. A computational fluid dynamics (CFD) model of the autoclave process was developed based on the finite volume method. The model predictions were compared with the measurements. Then, a control method was first used to improve the temperature field uniformity of a mold. The curing temperature difference (CTD) was proposed to describe the temperature field uniformity in the mold.

## 2. Experiment

### 2.1. Experimental Setup

**Figure 1:**
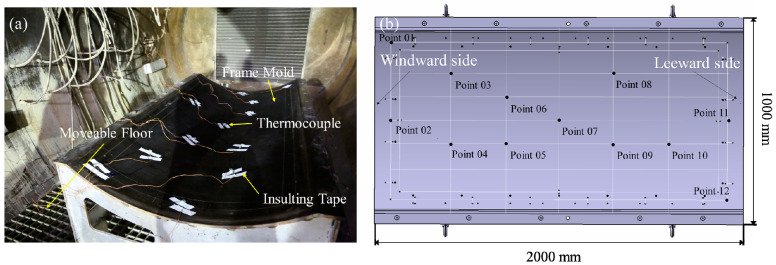
(**a**) The autoclave experiment setup, (**b**) a schematic of the mold with 12 measurement points.

The experiment was carried out in a full-scale industrial autoclave with a working area of 6 m in length and 3 m in diameter. Figure 1a shows the experimental setup. A frame mold was placed at the center of the moveable floor. The width, length, and height of the mold were 1, 2, and 0.5 m, respectively. The radius of the mold surface was 850 mm. Twelve thermocouples were fixed on the mold panel surface with white insulating tape. The thermocouple was the k type, and the calibration was ±1 °C. The temperature data were recorded every 120 s.

A schematic of the mold with 12 temperature measurement points is shown in Figure 1b. The positions of the 12 points were consistent with the positions of thermocouples in the experiment. The selected points covered the main area of the mold panel to characterize the temperature field in the mold. Points 01 and 02 were arranged at one end of the mold, which was close to the autoclave door called the windward side. Point 11 and point 12 were at the opposite side, which was called the leeward side, deeper into the autoclave.

**Figure 2:**
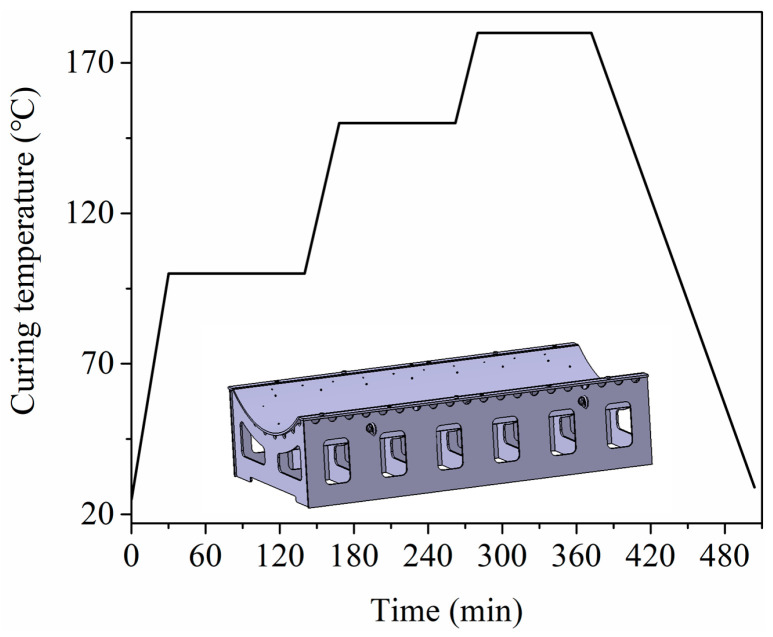
A typical curing cycle curve of AS4/8552.

Figure 2 shows a typical curing cycle curve of AS4/8552. It includes three constant temperature stages, and a decreasing temperature phase. The temperature increased from 25 to 100 °C during the ramping temperature stage, then the temperature remained at 100 °C for 110 min in the first stage. The second phase included the ramping temperature stage with a heating rate of 1.8 °C/min and a constant temperature stage with 150 °C. The maximum temperature reached 180 °C after the increasing temperature at the next stage. The last phase was the decreasing stage with a rate of 1.2 °C/min. The curing temperature was decided by the thermal property of composite materials. The pressure was set to 0.31 MPa in the experiment. 

### 2.2. Experimental Results 

**Figure 3:**
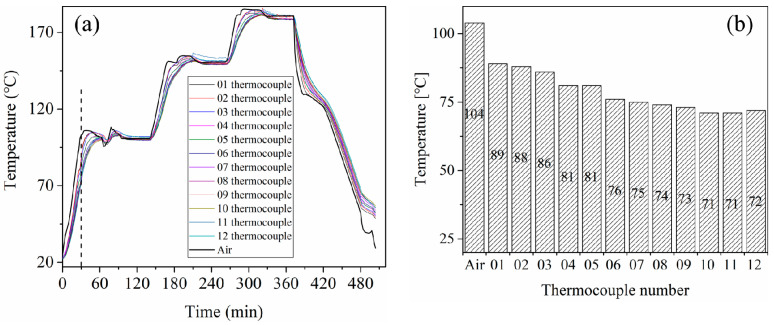
The temperature evolution of 12 points and the air temperature. (**a**) The entire process (**b**) at 30 min.

Figure 3a shows the temperature evolution of 12 measurement points and the air temperature in the autoclave. The air temperature was higher than the temperature of 12 points during the increasing temperature stage and lower than that of the 12 points during the decreasing temperature stage. The difference between the temperature of 12 points and air temperature was much smaller during the constant temperature stage.

Figure 3b illustrates the temperature values of all the points at 30 min, at which time the temperature difference was most significant. This is because the time of 30 min was the end of the first ramping temperature stage, after which time the temperature uniformity was significantly improved during the constant temperature stage. As shown in Figure 3b, the temperature of point 01 was the highest among all 12 points. The temperature of points at the windward side was higher than that at the leeward side. The lowest temperature points were 10 and 11. They were at the end of the leeward side and the lowest position of the curved panel. The maximum temperature difference among the 12 monitoring points was 18 °C. 

**Figure 4:**
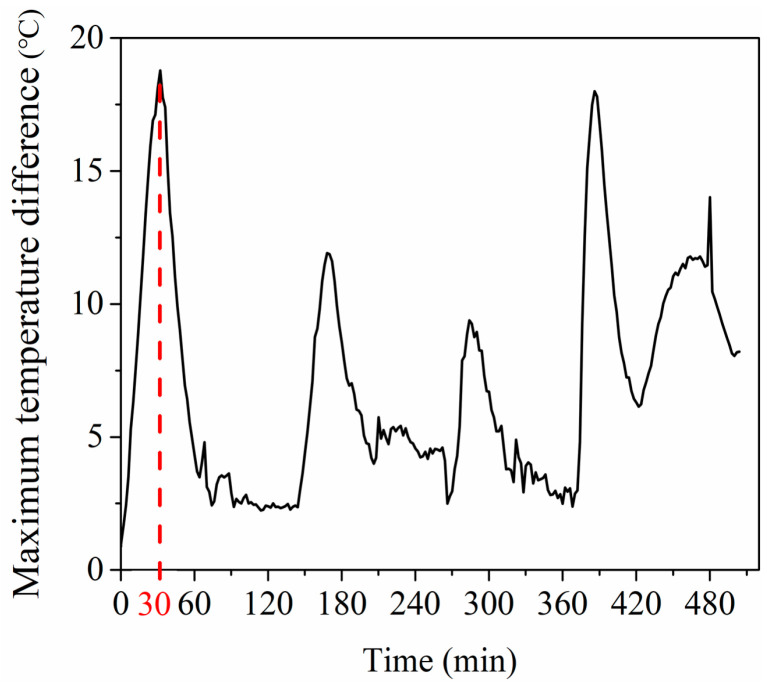
The temperature difference of 12 points.

Figure 4 shows the maximum temperature difference of 12 measurement points during the whole curing process. As can be seen from Figure 4, the temperature difference of 12 measurement points experienced several large fluctuations, and these time periods corresponded to the increasing temperature stage. The temperature difference peak was 18 °C at 30 min. On the other hand, in the constant temperature stages, the temperature difference value as well as the fluctuation were small.

## 3. Numerical Model

**Figure 5:**
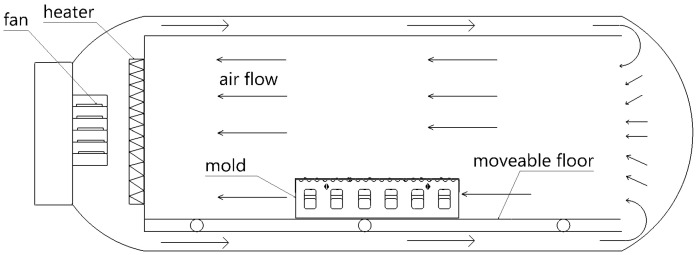
The working principle of autoclave.

The working principle of an autoclave is shown in Figure 5. A fan accelerates the heating air circulation in the autoclave. The heater heats air to a desired temperature. The heated airflow direction is shown by the arrows. The heated airflow is transported from the autoclave door side into the interior. The tools and composite part are heated by the circulating hot airflow.

### 3.1. Convective Heat Transfer Model

According to the working principle of autoclave, a convective heat transfer model was developed to simulate the heat transfer process of the autoclave. The heat transfer was expressed by three physical laws in the fluid domain: Mass conservation, momentum conservation, and energy conservation [26].

Mass conservation equation:

(1) $$\frac{\partial \rho }{\partial t}+div\left(\rho U\right)=0$$

Momentum conservation equation:

(2) $$\frac{\partial (\rho U)}{\partial t}+div(\rho U⊗U)=div(\mu \;grad\;U)-grad\;p+S$$

where *ρ* is the fluid density, *μ* is the fluid dynamic viscosity, *p* is the fluid pressure, *S* is the generalized source term of the momentum equation, and **U** is the component of velocity in x, y, and z directions.

Based on the law of conservation of energy, the energy equation can be expressed with fluid enthalpy *h* and temperature *T*, when thermal Fourier law is introduced.

Energy conservation equation:

(3) $$\frac{\partial (\rho h)}{\partial t}+div(\rho hU)=div\left(\lambda \;grad\;T\right)-pdiv(U)+S_{h}+\varphi$$

where *h* = *h* (*p,T*) is the convection heat coefficient, *λ* is the thermal conductivity of the fluid, *S_h_* is the internal heat source of fluid, *φ* is the part of electrical energy transformed by mechanical energy due to viscous dissipation; and *T* is fluid temperature. The equation system composed of the above formulas cannot be a closed system, so two gas state equations need to be added.

Gas state equation:

(4) ρ=f(p,T)

In the solid region, the governing equation of heat conduction in the tool is given by:

(5) $$\rho_{s}c_{s}\frac{\partial \left(T\right)}{\partial t}=div\left(\lambda_{s}div\left(T\right)\right)+S_{T}$$

where *ρ_s_* is the density of solid, *C_s_* is the specific heat of solid, *λ_s_* is the thermal conductivity of the solid, *T* is the temperature of solid, and *S_T_* is the internal heat generation.

The heating process of mold is realized through a forced convection of fluid, so the type of flow has to be considered. The flow type includes laminar flow and turbulent flow, and it can be defined by the Reynolds number (*R_e_*):

(6) $$R_{e}=\frac{\rho \nu_{m}D}{\mu }$$

where *ρ* is the fluid density, *ν_m_* is the average velocity of the fluid, *D* is the hydraulic diameter, and *μ* is the fluid velocity. The flow in the autoclave is defined as turbulent flow when the value of *R_e_* is higher than 12,000 [23]. The following parameters were applied in the current paper: *ρ* = 1.24 kg/m^3^, *μ* = 17.9 × 10^−6^ kg/m·s, *ν_m_* = 4 m/s, and *D* = 3 m, which is the diameter of the autoclave. According to Equation (6), *R_e_* = 8.3 × 10^5^ > 12,000. Therefore, the flow state of the gas is turbulent flow.

### 3.2. Geometric Model and Mesh

Based on the convective heat transfer model proposed in the previous section, a finite volume method was applied to simulate the temperature field of the mold using the Fluent (version 16.0). Considering the complex geometry of mold, the RANS turbulence model was applied to the fluid region in the simulation. The K-Epsilon turbulence sub-model was selected due to the model accuracy and computing cost.

**Figure 6:**
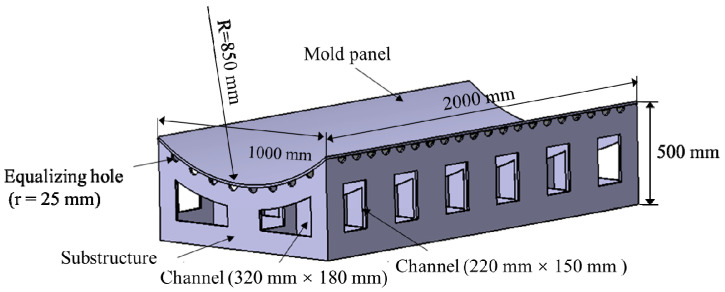
Simplified mold model.

The simplified mold geometric model was used in the simulation, as shown in Figure 6. The mold consisted of a substructure and a mold panel. The substructure was made of some support grids with equalizing hole and channel, which allowed air to pass through the substructure uniformly. The thicknesses of the mold panel and support grids were 15 and 10 mm, respectively. The radius of each equalizing hole was 25 mm, and the approximate dimensions of two different channels were 220 × 150 mm and 320 × 180 mm, respectively. 

**Figure 7:**
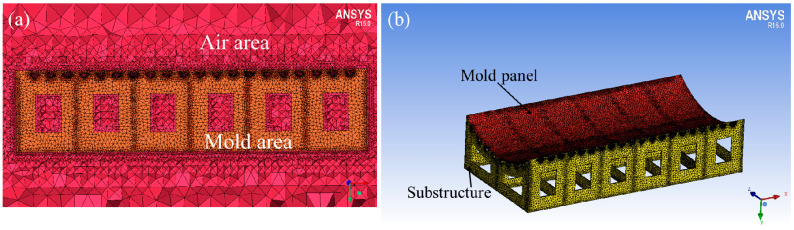
(**a**) The side view of fluid-solid conjugated mesh interface, (**b**) the mesh of mold.

An unstructured mesh was selected due to the complex mold structure. During the process of fluid-solid conjugated heat transfer, the mesh condition of the fluid-solid conjugated interface influences the accuracy of the simulation. As shown in Figure 7a, the density of grids gradually decreased from the solid area (yellow) to fluid area (red) to improve the accuracy of the calculation. The mesh of the mold is shown in Figure 7b. The mold was made of Q235-A steel (Fe 360A), having the density of 8030 kg/m^3^, specific heat of 502 J/kg/K, and thermal conductivity of 16.2 w/m/K.

### 3.3. Boundary Conditions and Solvers

**Figure 8:**
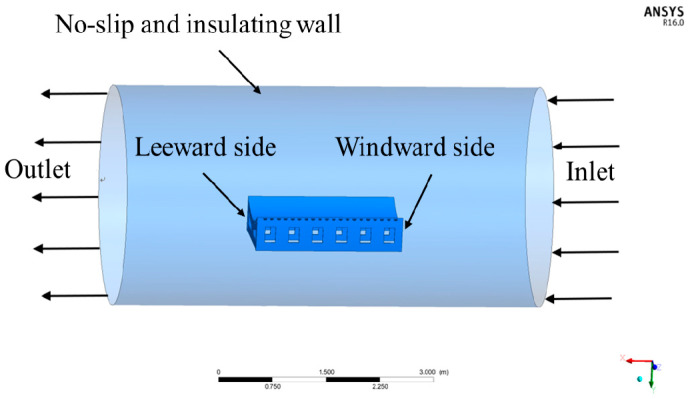
Boundary conditions of the autoclave.

According to the actual conditions of the autoclave process, the simulation model was simplified as shown in Figure 8. The effective calculation area of the autoclave was an inner cavity with a cylindrical space. The surface of the autoclave was a no-slip and insulating wall [27]. The inlet temperature boundary condition was defined as the curing temperature, as shown in Figure 3. The temperature profile was loaded by the user-defined functions (UDFs) file. The UDFs file was performed to achieve the change of air temperature at the inlet in Fluent. The average velocity of the inlet was defined as 4 m/s [28]. The outlet was characterized by an outflow condition, which was applied when the flow velocity and pressure distribution at the outlet were unclear. A pressure-based solver and an implicit algorithm were used to solve the equations. The defined criterion for convergence was the residual value lower than 10^−5^ for the applied equations [29].

### 3.4. Validation of the Numerical Model

**Figure 9:**
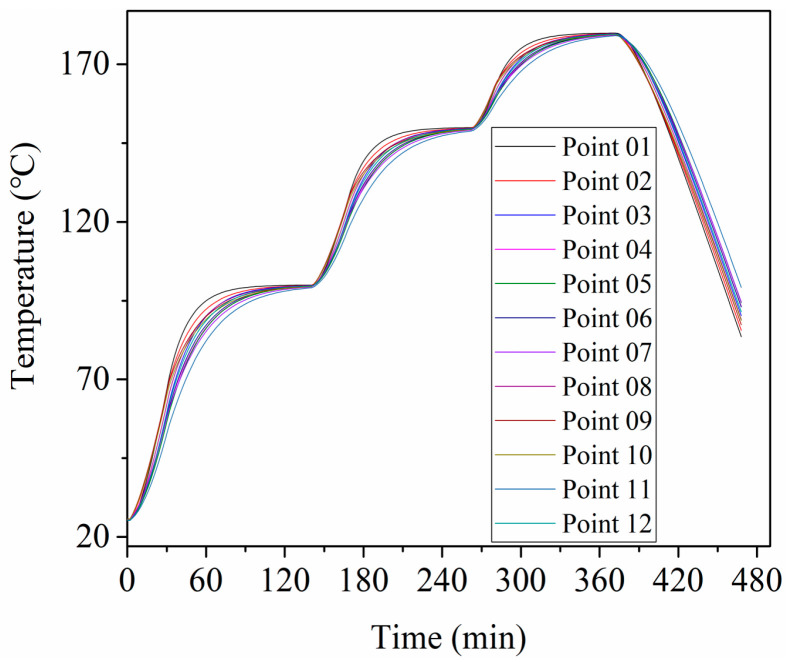
The simulation results of 12 points.

**Figure 10:**
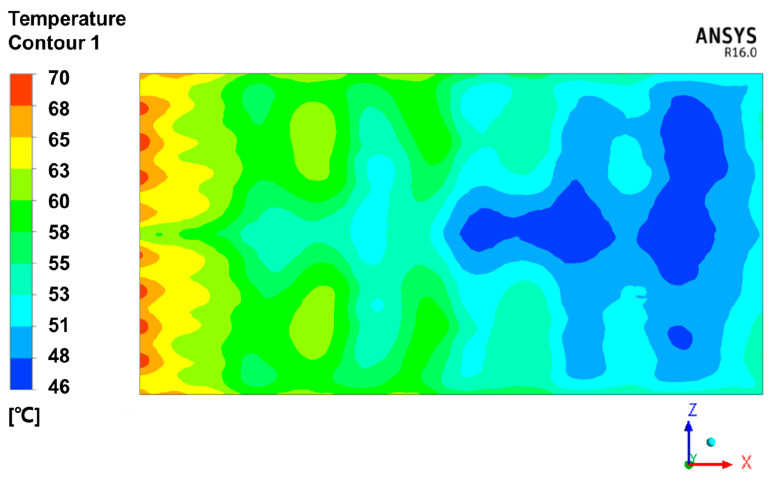
The temperature contour plot of the mold panel surface at 30 min.

Figure 9 shows the simulation results of 12 points, which correspond to the 12 measurement points in the experiment. It can be seen from Figure 9 that the temperature evolution of 12 points was basically the same. Figure 10 shows the temperature contour plot of the mold panel surface at 30 min. The temperature field on the windward side was obviously higher than that on the leeward side area. This is because the circulation of hot air was blocked by the substructure, which influenced the efficiency of convective heat transfer.

**Figure 11:**
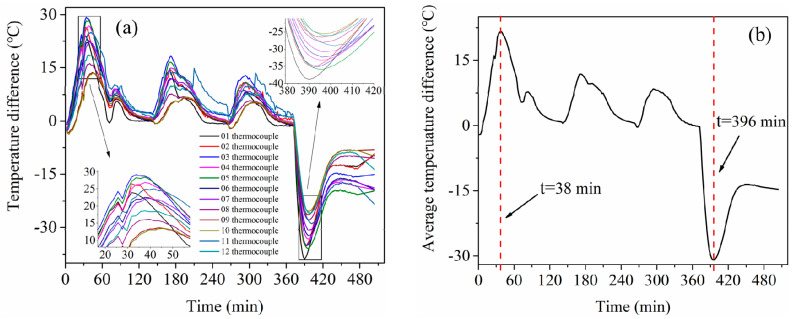
The temperature difference of 12 points between simulation and experiment. (**a**) The difference values, (**b**) the average difference.

To compare the numerical and experimental results, the difference between them was calculated, as illustrated in Figure 11a. The evolution of the difference values was basically the same for all the 12 points. The difference values were relatively large at the periods of 25–50 min and 380–420 min. The average temperature difference of 12 points is presented in Figure 11b. The maximum average difference during the temperature rising stage was 21.7 °C at 38 min. The maximum relative difference between numerical and experimental results was 5.9% during temperature increasing and constant stages, which is close to the result of 5% in literature [23]. The maximum average difference during the cooling stage was 30.1 °C at 396 min, as shown in Figure 11.

**Figure 12:**
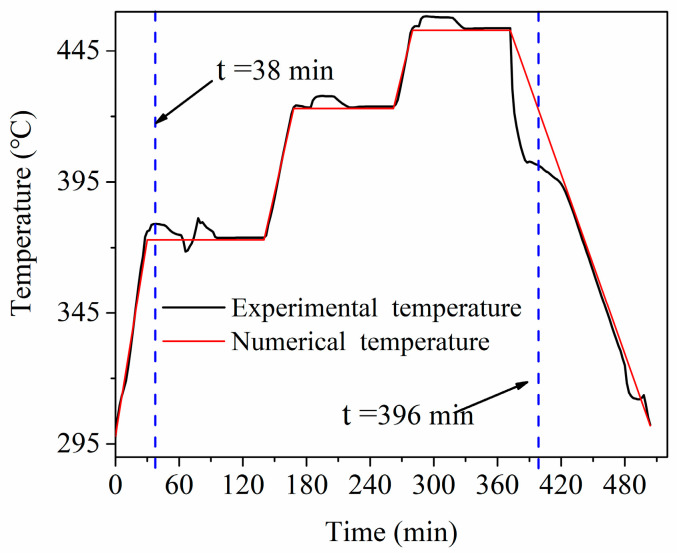
The air temperature of simulation and experiment.

Figure 12 shows the evolution of air temperature used in the simulation and experiment. At each temperature rising stage, the deviation was very small. In the constant temperature stage and the decreasing temperature stage, the air temperature experienced some fluctuations. Especially at the beginning of the cooling stage, the deviation was large. This may be one of the reasons for the discrepancy between the predicted and measured temperature history for the 12 points in Figure 11. Moreover, the assumptions made in the model for the boundary conditions might also lead to the discrepancy. The third reason may be the measurement error in the experiment. 

## 4. Control of Temperature Distribution in the Mold Panel

### 4.1. Research Concept

The maximum temperature difference of the mold was 18 °C at the first rising temperature stage, as shown in Figure 4. The temperature gradient of the mold would increase the uncertainty of the curing process. Decreasing the temperature difference can ensure high precision as well as curing quality of parts. The standard deviation (SD) was used to evaluate the temperature field uniformity in the mold at the first stage. The standard deviation was calculated by collecting 1000 points evenly distributed in the mold panel using the CFD-post software. Note that the standard deviation at 30 min will be discussed in the following part because the temperature difference was the most significant at this moment.

### 4.2. Methodology of Temperature Distribution Control 

**Figure 13:**
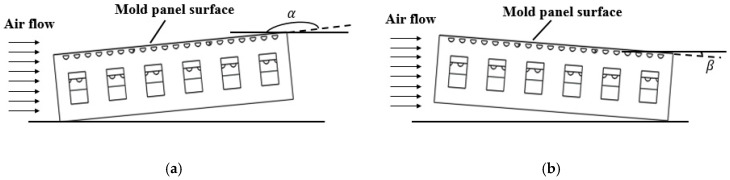
Two types of tilt methods. (**a**) Type 1, (**b**) Type 2.

Figure 13 illustrates the method for controlling the temperature distribution by changing the placement angle of the mold. Two tilt methods were studied in this paper. The inclination angle of mold should not be too large in order to prevent the surplus resin flowing from the high side to the low side. Therefore, the value of α was selected as 175, 170, and 168°, respectively, and the value of β was selected as 5, 10, and 12°, respectively.

With the optimized placement angle of the mold, the air was injected at a certain angle onto the mold surface. The heat transfer efficiency was improved due to the shorter flow distance of the fluid and the thinner flow boundary layer [30]. The high heat transfer efficiency made the temperature distribution uniform. Therefore, the SD value was reduced.

### 4.3. Results and Discussion

**Figure 14:**
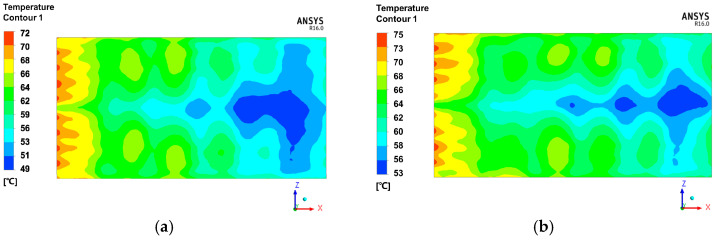

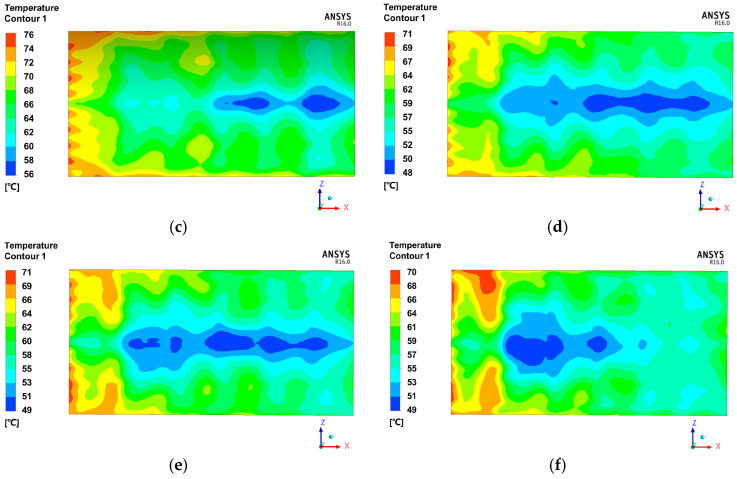
Temperature distribution contours of different angles. (**a**) α = 175°, (**b**) α = 170°, (**c**) α = 168°, (**d**) β = 5°, (**e**) β = 10°, (**f**) β = 12°.

Figure 14a–f presents the temperature contour plot in the mold panel surface at 30 min for different placement angles of the mold. The high-temperature area was located on the side of the air inlet for all placement angles of the mold. Both the maximum and minimum temperatures increased with the decrease in α. This is due to the improved heat transfer efficiency by the field synergy (coordination) principle [30]. The temperature value in the mold had no obvious change when the angle β was increased. However, the position of low-temperature area transferred from the leeward side to windward side, possibly because the substructure of mold blocked the air flow. Therefore, changing α is an effective method to improve the uniformity of temperature distribution.

Table 1 shows the SD values and the maximum temperature difference in the mold panel surface when α was equal to 180, 175, 170, and 168°. The maximum temperature difference gradually decreased with the decrease in α. The SD decreased by 13.3% as the value of α changed from 180 to 168°. This means a more uniform temperature field can be achieved by decreasing the placement angle α.

## 5. Conclusions

In the present study, experiments on temperature distribution in a frame mold panel were performed. Then, a numerical model of the autoclave process was developed by using the finite volume method. A method of controlling the temperature distribution was finally proposed. The following conclusions were drawn:

1. The maximum temperature difference of 18 °C for the 12 measurement points appeared at the timing of 30 min.
2. The difference between the predicted temperature and the experimental temperature was 5.92%, which shows a good agreement.
3. The proposed controlling method can significantly improve the temperature uniformity in the mold. The standard deviation decreased by 13.3% as the placement angle α changed from 180 to 168°.

## Figures and Tables

**Table 1:** The standard deviation (SD) and the maximum temperature difference in the mold panel surface.

α (°)	The Maximum Difference (°C)	SD (°C)
180	24.1	5.71
175	22.8	5.21
170	22.2	5.01
168	19.9	4.95
